# False aminoglycoside resistance in Enterobacterales and non-fermenters by an automated testing system: a descriptive study

**DOI:** 10.1128/spectrum.03093-23

**Published:** 2023-11-16

**Authors:** P. M. C. Klein Klouwenberg, W. Altorf–van der Kuil, A. J. van Griethuysen, M. Hendriks, E. J. Kuijper, D. W. Notermans, A. F. Schoffelen, on behalf of the ISIS-AR study group, J.W.T. Cohen Stuart

**Affiliations:** 1 Department of Medical Microbiology, Noordwest Ziekenhuisgroep, Alkmaar, the Netherlands; 2 Department of Medical Microbiology, Meander Medical Center, Amersfoort, the Netherlands; 3 Department of Medical Microbiology and Infection Prevention, Amsterdam UMC, University of Amsterdam, Amsterdam Infection and Immunity Institute, Amsterdam, the Netherlands; 4 Department of Medical Microbiology, Atalmedial, Amsterdam, the Netherlands; 5 Department of Medical Microbiology, OLVG Lab BV, Amsterdam, the Netherlands; 6 Public Health Service, Public Health Laboratory, Amsterdam, the Netherlands; 7 Department of Medical Microbiology and Infection Prevention, Gelre Hospitals, Apeldoorn, the Netherlands; 8 Centre for Infectious Disease Control (CIb), National Institute for Public Health and the Environment (RIVM), Bilthoven, the Netherlands; 9 Microvida Amphia, Laboratory for Microbiology and Infection Control, Breda, the Netherlands; 10 Department of Medical Microbiology, IJsselland Hospital, Capelle aan den IJssel, the Netherlands; 11 Department of Medical Microbiology, Haeseker, Reinier de Graaf Group, Delft, the Netherlands; 12 Department of Medical Microbiology, Deventer Hospital, Deventer, the Netherlands; 13 Department of Medical Microbiology, Slingeland Hospital, Doetinchem, the Netherlands; 14 Department of Medical Microbiology, Albert Schweitzer Hospital, Dordrecht, the Netherlands; 15 Department of Medical Microbiology, Gelderse Vallei Hospital, Ede, the Netherlands; 16 Department of Medical Microbiology, Admiraal De Ruyter Hospital, Goes, the Netherlands; 17 Department of Medical Microbiology and Infection Prevention, Groene Hart Hospital, Gouda, the Netherlands; 18 Department of Medical Microbiology, Haaglanden MC, 's-Gravenhage, the Netherlands; 19 Department of Medical Microbiology, Haga Hospital, 's-Gravenhage, the Netherlands; 20 Certe, Medical Microbiology Groningen|Drenthe, Groningen, the Netherlands; 21 Department of Medical Microbiology, University of Groningen, University Medical Center, Groningen, the Netherlands; 22 Regional Public Health Laboratory Haarlem, Haarlem, the Netherlands; 23 Department of Medical Microbiology, St Jansdal Hospital, Harderwijk, the Netherlands; 24 Department of Medical Microbiology and Infection Control, Jeroen Bosch Hospital, 's-Hertogenbosch, the Netherlands; 25 Department of Medical Microbiology, CBSL, Tergooi MC, Hilversum, the Netherlands; 26 Certe, Medical Microbiology Friesland|NOP, Leeuwarden, the Netherlands; 27 Department of Medical Microbiology, Leiden University Medical Center, Leiden, the Netherlands; 28 Department of Medical Microbiology, Eurofins Clinical Diagnostics, Leiden-Leiderdorp, the Netherlands; 29 Department of Medical Microbiology, Maastricht University Medical Centre, Maastricht, the Netherlands; 30 Department of Medical Microbiology and Immunology, St Antonius Hospital, Nieuwegein, the Netherlands; 31 Department of Medical Microbiology and Infectious Diseases, Canisius Wilhelmina Hospital, Nijmegen, the Netherlands; 32 Department of Medical Microbiology, Radboud University Medical Center, Nijmegen, the Netherlands; 33 Laurentius Hospital, Roermond, the Netherlands; 34 Department of Medical Microbiology, Bravis Hospital, Roosendaal, the Netherlands; 35 Department of Medical Microbiology and Infectious Diseases, Erasmus University Medical Center, Rotterdam, the Netherlands; 36 Department of Medical Microbiology and Infection Control, Franciscus Gasthuis and Vlietland, Rotterdam, the Netherlands; 37 Department of Medical Microbiology, Ikazia Hospital, Rotterdam, the Netherlands; 38 Star-SHL, Rotterdam, the Netherlands; 39 Department of Medical Microbiology and Infection Control, Zuyderland Medical Centre, Sittard-Geleen, the Netherlands; 40 Department of Medical Microbiology, Microvida ZorgSaam, Terneuzen, the Netherlands; 41 Department of Medical Microbiology, Microvida ZorgSaam, Terneuzen, the Netherlands; 42 Department of Medical Microbiology, St. Elisabeth Hospital, Tilburg, the Netherlands; 43 Department of Medical Microbiology and Immunology, Diakonessenhuis, Utrecht, the Netherlands; 44 Department of Medical Microbiology, Saltro Diagnostic Centre, Utrecht, the Netherlands; 45 Department of Medical Microbiology, University Medical Center Utrecht, Utrecht, the Netherlands; 46 Department of Medical Microbiology, Eurofins-PAMM, Veldhoven, the Netherlands; 47 Rijnstate Hospital, Laboratory for Medical Microbiology and Immunology, Velp, the Netherlands; 48 Department of Medical Microbiology, VieCuri Medical Center, Venlo, the Netherlands; 49 Isala Hospital, Laboratory of Medical Microbiology and Infectious Diseases, Zwolle, the Netherlands; 1 Department of pharmacy, Fundashon Mariadal, Kralendijk, Bonaire, the Netherlands; 2 Centre for Infectious Disease Control (CIb), National Institute for Public Health and the Environment (RIVM), Bilthoven, the Netherlands; 3 Department of Medical Microbiology, Gelderse Vallei Hospital, Ede, the Netherlands; JMI Laboratories, North Liberty, Iowa, USA

**Keywords:** antimicrobial susceptibility testing, gentamicin, aminoglycosides, automated testing system

## Abstract

The majority of antimicrobial susceptibility testing (AST) in Dutch medical microbiology laboratories (MMLs) is performed using the automated system VITEK2 or Phoenix. One of the Phoenix users noted a high percentage of gentamicin resistance in *Proteus mirabilis* compared to national resistance data. We therefore performed a descriptive analysis comparing gentamicin and tobramycin minimum inhibitory concentration (MIC) distributions for Enterobacterales and non-fermenters as measured using VITEK2 and Phoenix. Routine susceptibility data from 5 MMLs using Phoenix and 31 using VITEK2 with complete data in the Dutch national surveillance system for antimicrobial resistance from January 2016 to December 2021 were included. A panel of 12 discrepant isolates was sent to Becton Dickinson for confirmation. In general, Phoenix measured higher MICs, with discrepancies being most prevalent for *Proteus*, *Providencia*, *Morganella*, *Serratia,* and *Acinetobacter* species (borderline susceptibility for gentamicin ranging from 1% for VITEK2 to 67% for Phoenix systems), and less, but still clinically relevant, for *Escherichia coli* (1%–12%). Furthermore, we observed a yearly increase in resistance for *Proteus* and *Providencia* species measured by Phoenix. Similar discrepancies were found for tobramycin. The company confirmed our findings on all strains. Significantly more false aminoglycoside borderline susceptible and resistant Enterobacterales were found using Phoenix compared to the VITEK2 system. These findings should be taken into consideration in the development of clinical treatment guidelines for patients with sepsis.

**IMPORTANCE:**

Antimicrobial sensitivity data are important to guide antimicrobial therapy. In microbiological laboratories, routine sensitivity measurements are typically performed with automated testing systems such as VITEK2 and Phoenix. Using data from the Dutch national surveillance system for antimicrobial resistance over a 6-year period, we found that the measured minimum inhibitory concentrations for aminoglycosides in Enterobacterales and non-fermenters were too high for the Phoenix system. In addition, we observed a yearly increase in resistance for several species measured by Phoenix. These findings might have consequences for clinical treatment of patients with sepsis.

## INTRODUCTION

Aminoglycosides are important components of the empirical treatment of sepsis, most frequently in combination with other antibiotics (1, 2). For Enterobacterales, the European Committee on Antimicrobial Susceptibility Testing (EUCAST) recommends a clinical breakpoint of 2 mg/L for gentamicin and tobramycin, irrespective of systemic or urinary tract infections (3). For *Pseudomonas aeruginosa*, only tobramycin is recommended with a similar breakpoint as Enterobacterales. *Acinetobacter* species have a higher breakpoint of 4 mg/L.

In a recent study from the Netherlands, most Enterobacterales species from positive blood cultures taken at the emergency department were tested susceptible to gentamicin using the Phoenix automated testing system, but 9% of Enterobacterales had exactly a minimum inhibitory concentration (MIC) of 2 mg/L, which is at the breakpoint and clinically considered as “borderline susceptible” (4). The authors concluded that the desired pharmacokinetic parameter (ratio between the peak serum concentration and the minimum inhibitory concentration, or Cmax/MIC, of 8–10) could not be achieved using a standard dose of 5 mg/kg in patients with these borderline susceptible isolates. Therefore, they advised a daily dose of 7 mg/kg, which is in accordance with EUCAST dosage recommendations (3).

The majority of antimicrobial susceptibility testing (AST) in Dutch medical microbiology laboratories (MMLs) is performed using the automated system VITEK2 or Phoenix. One of the MMLs that used Phoenix noted a high percentage of gentamicin resistance in *Proteus mirabilis* compared to aggregated national resistance data (5). Since differences in aminoglycoside MIC distributions could have implications for sepsis treatment, we performed a descriptive analysis comparing gentamicin and tobramycin MIC distributions for Enterobacterales and non-fermenters as measured using VITEK2 and Phoenix automated AST systems.

## MATERIALS AND METHODS

We extracted data from the Dutch national surveillance system for antimicrobial resistance (ISIS-AR), which is based on data from Dutch MMLs (6). Ethical approval and consent were not needed for the study, since it is based on surveillance data only. Data from 36 MMLs with complete data from January 2016 to December 2021 were included. Five of these MMLs used Phoenix (Becton, Dickinson and Company [BD], Franklin Lakes, NJ, USA; further referred to as Phoenix MMLs), and 31 MMLs used VITEK2 (BioMérieux, Marcy l’Etoile, France; further referred to as VITEK2 MMLs). No other automated systems with complete data for the whole period were used in the Netherlands. We extracted MIC values of gentamicin and tobramycin in the first diagnostic isolate per patient per species per year of Enterobacterales (*Citrobacter freundii, Escherichia coli, Enterobacter cloacae* complex*, Klebsiella aerogenes, Klebsiella pneumoniae, Morganella morganii, P. mirabilis, Providencia* species*,* and *Serratia marcescens*), *Acinetobacter* species, and *P. aeruginosa*. We excluded data on amikacin, since insufficient data were available. MIC values were re-interpreted according to EUCAST breakpoints version 12.0 (2022), with borderline susceptibility defined as MIC 2 mg/L for Enterobacterales and *P. aeruginosa* (tobramycin only) and MIC 4 mg/L for *Acinetobacter* species, and low-level resistance as one MIC-step above the breakpoints. The prevalence of borderline susceptible and resistant isolates using Phoenix and VITEK2 was compared, both overall and on species levels, in isolates from all specimens together and those from blood cultures only. We repeated the analysis for third-generation cephalosporin-resistant isolates, including extended-spectrum beta-lactamase-producing isolates, since aminoglycosides are especially useful for patients infected with such pathogens. Data were analyzed using R (version 4.2.2) including the calculation of 95% confidence intervals.

## RESULTS

In total, gentamicin susceptibility testing results were available for 1,285,013 VITEK2 isolates and 230,904 Phoenix isolates, and tobramycin testing results for 1,288,777 VITEK2 and 158,193 Phoenix isolates. For blood isolates, 48,818 gentamicin testing results were available from VITEK2 and 8,545 from Phoenix, and 48,826 tobramycin results from VITEK2 and 8,483 from Phoenix. *E. coli* was the most isolated pathogen (65%), followed by *K. pneumoniae* (10%), *P. mirabilis* (8%), and *P. aeruginosa* (8%). Species distribution was similar between Phoenix and VITEK2 laboratories.

### Differences in MIC distributions

**Fig 1:**
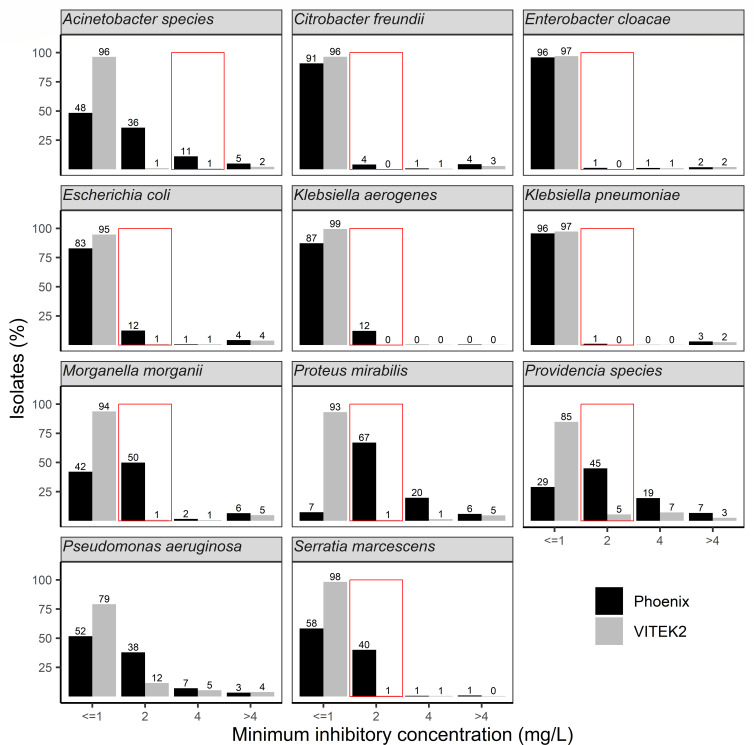
Minimal inhibitory concentrations for gentamicin as measured by Phoenix (black bars) and VITEK2 (gray bars) in all clinical isolates between 2016 and 2021. The red box indicates borderline susceptible isolates (minimum inhibitory concentration = 2 mg/L), except for *Acinetobacter* species (4 mg/L) and *Pseudomonas aeruginosa* (no EUCAST breakpoints for gentamicin).

Figure 1 shows the MIC distribution for gentamicin in all isolates. Using the EUCAST criteria, 3.8% of Enterobacterales were resistant to gentamicin by VITEK2 and 8.3% by Phoenix. Significant differences in resistance to gentamicin were found for *P. mirabilis* (6.0% vs 25%) and *Providencia* species (9.7% vs 26%), respectively. Low-level resistance to gentamicin was present in 1.0% and 2.8% of isolates tested with VITEK2 compared to Phoenix, respectively. Again, differences were more pronounced for certain species, e.g., *P. mirabilis* (1% vs 20%) and *Providencia* (7% vs 19%, respectively). Borderline susceptibility to gentamicin was present in 1.6% (95% confidence interval 1.6–1.6) of isolates measured by VITEK2, but 18.8% (18.7–19.0) when measured by Phoenix. This difference was most evident for *P. mirabilis* (1% vs 67%), *S. marcescens* (1% vs 40%), *M. morganii* (1% vs 50%), and *Providencia* species (5% vs 45%), and less (but still significant) for *E. coli* (1% vs 12%), *K. aerogenes* (0% vs 12%), and *Acinetobacter* species (1% vs 11%). No clinically significant differences were observed for *C. freundii, E. cloacae,* and *K. pneumoniae*.

**Fig 2:**
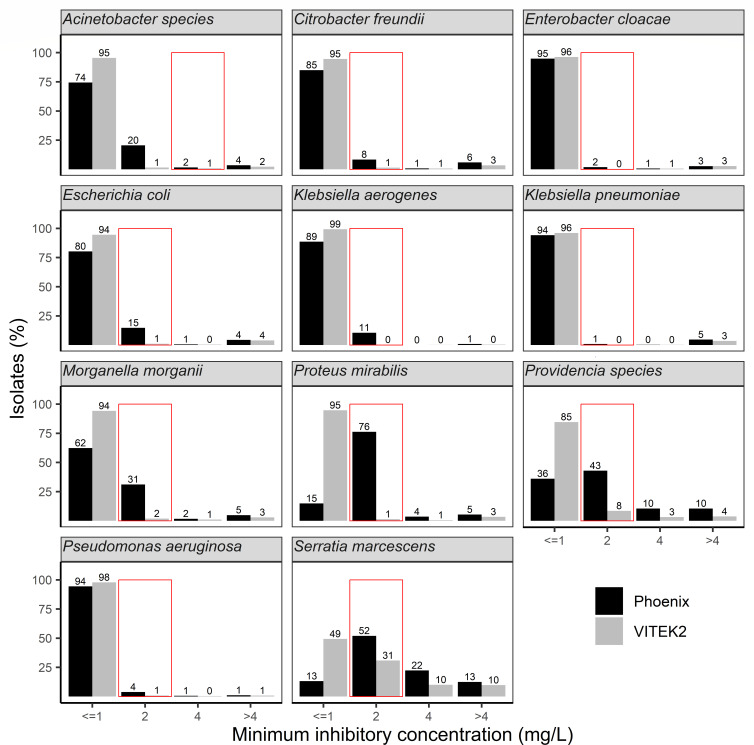
Minimal inhibitory concentrations for tobramycin as measured by Phoenix (black bars) and VITEK2 (gray bars) in all clinical isolates between 2016 and 2021. The red box indicates borderline susceptible isolates (minimum inhibitory concentration = 2 mg/L), except for *Acinetobacter* species (4 mg/L).

Figure 2 shows a similar MIC distribution for tobramycin in all isolates. Observed trends were comparable to those for gentamicin, with a percentage of borderline susceptible isolates of 1.5% (1.5%−1.6%) versus 17.5% (17.3%−17.7%) for VITEK2 and Phoenix, respectively. Again, the largest differences were seen in *P. mirabilis*, *M. morganii,* and *Providencia*. For *S. marcescens*, however, the difference between both systems was smaller than that for gentamicin because the VITEK2 system also measured slightly higher levels of borderline susceptibility.

Results for both gentamicin and tobramycin were similar when focusing on third-generation cephalosporin-resistant isolates (4.5% of isolates were cephalosporin-resistant when measured with VITEK2 compared to 4.8% using Phoenix), with higher levels of borderline susceptibility for *P. mirabilis* (53% vs 2%), *S. marcescens* (39% vs 3%), *M. morganii* (52% vs 2%), *Providencia* species (54% vs 4%), and *K. pneumoniae* (40% vs 1%), and a smaller difference for *E. coli* (14% vs 2%) when measured with Phoenix compared to VITEK2.

### Shifts of MIC distributions over time

**Fig 3:**
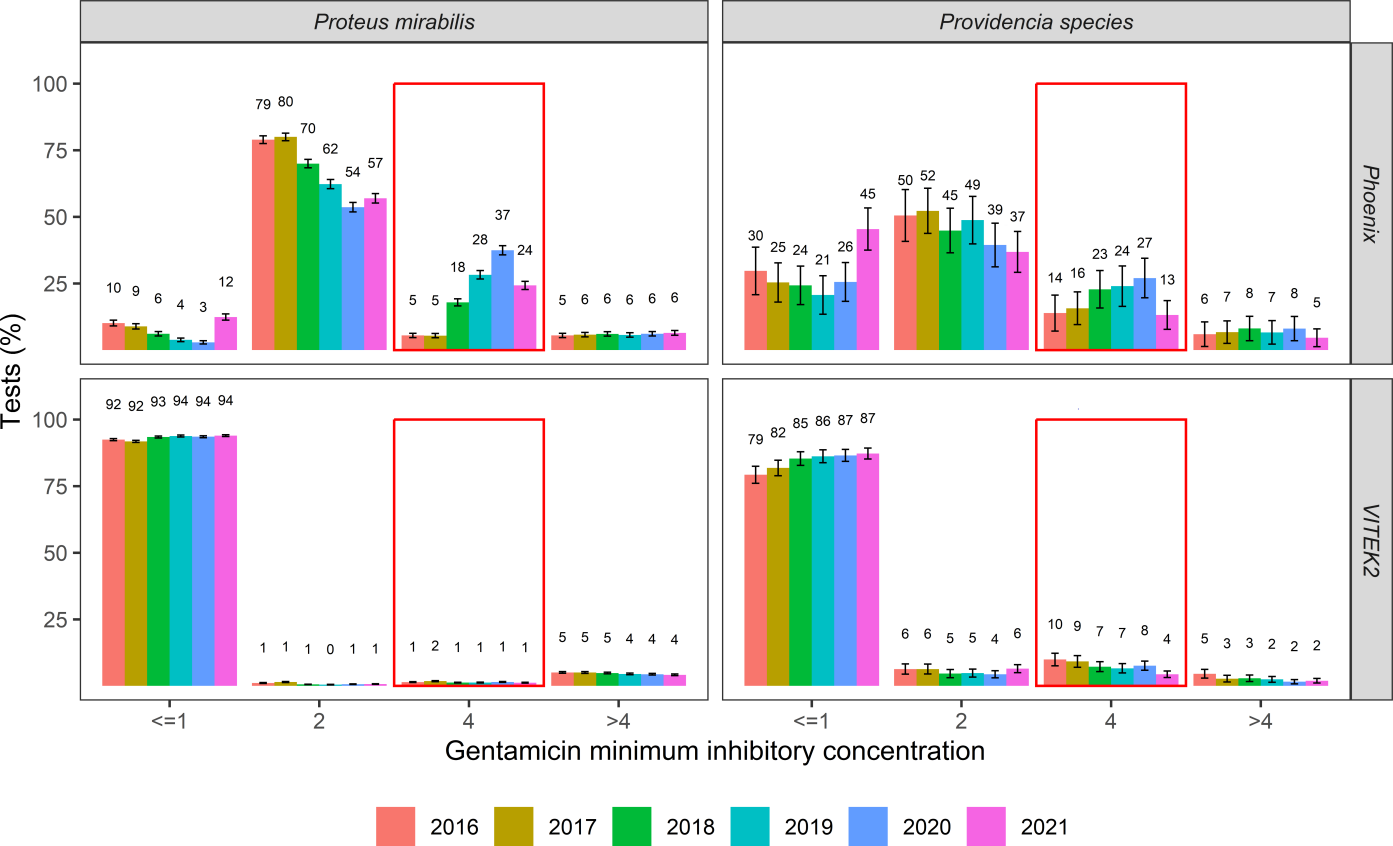
Yearly gentamicin MIC distributions for *Providencia* and *P. mirabilis* isolates. The proportion of “borderline” resistant isolates (minimum inhibitory concentration 4 mg/L) increases yearly from 2016 to 2020 for *P. mirabilis* and *Providencia* species but not for other species (data not shown).

Figure 3 shows the yearly gentamicin MIC distributions for *P. mirabilis* and *Providencia* isolates. The percentage of isolates with “low level” resistance increased over time in Phoenix MMLs from 2016 to 2020: from 5% in 2016 to 37% in 2020 in *P. mirabilis* and from 14% to 27% in *Providencia* species. This increase in resistance seemed to be caused by a shift from borderline susceptible isolates to more resistant isolates. This phenomenon was not observed nationwide, since the measured gentamicin MIC values in isolates from VITEK2 MMLs remained stable over time. Interestingly, in 2021, the percentage of resistance in *Proteus* and *Providencia* species decreased. No time trends were found for other Enterobacterales, *Acinetobacter* species*,* and *P. aeruginosa*.

## DISCUSSION

We observed clinically important differences between the Phoenix and VITEK2 automated AST systems in gentamicin and tobramycin MIC distributions of diagnostic Enterobacterales isolates. In general, the Phoenix system measured higher MICs as compared to VITEK2. Differences in the percentage of borderline susceptible isolates were especially apparent for *Proteus*, *Morganella*, and *Serratia* species, and less, but still clinically relevant, for *E. coli*. Furthermore, we observed a yearly increase up to 2020 in gentamicin MIC values and low-level resistance for *Proteus* and *Providencia* species measured by Phoenix, but not by VITEK2.

This analysis was a follow-up of an observation made in 2020, when a Phoenix user in the Netherlands contacted the national surveillance for antimicrobial resistance at the Dutch national institute of public health (RIVM) to report a higher gentamicin resistance as measured in that laboratory among *Proteus* and *Providencia* species compared to national resistance data. Twelve *Proteus* strains were selected and sent to BD for confirmation on a Phoenix M50 with NMIC‐500 cards. In addition, BD compared the current performance of their panels against an established test battery with known

historic MICs and a second set consisting of 75 Enterobacterales isolates and 75 non-fermenters. The conclusion was that indeed false gentamicin resistance occurred, and the concentration of gentamicin in Phoenix cards was subsequently increased since April 2022 (personal communication with the Senior Quality Manager, BD, 6 August 2022). However, it remains unclear whether Phoenix panels other than NMIC-408 and UNMIC-409 were updated. Future studies are necessary to ensure that these changes have solved the issue of increased MICs for aminoglycosides.

Gentamicin susceptibility testing in automated systems has been standardized for medium composition, inoculum size, and duration of the incubation. No information is available on the precise medium composition of each manufacturer. Speculating on the falsely elevated MICs for aminoglycosides for several species, an additional literature search revealed interesting data describing that cation concentrations, osmolarity, and pH changes significantly affect the MIC values of aminoglycosides for several bacterial species, which should be studied in more detail (7 – 9).

There is a lot of debate around the role and optimal dosing of aminoglycosides in the treatment of patients with sepsis. In this light, our findings of a difference between the measurements of automated systems are relevant and should be taken into consideration in the development of sepsis treatment guidelines. A standard gentamicin dosage of 5 mg/kg body weight will not reach sufficient high plasma levels in patients suffering infections with Enterobacterales, which are borderline susceptible to aminoglycosides, and a standard dose of 7 mg/kg was therefore recently proposed (1). However, based on the lower MIC values as measured by VITEK2, we conclude that the lower dosage of 5 mg/kg of gentamicin might be sufficient in the majority of patients with sepsis since ~95% of tested strains had a gentamicin MIC ≤ 1 mg/L, and therefore, the optimal pharmacokinetic parameter of 8–10 times the Cmax/MIC should be achieved using a dosage of 5 mg/kg in almost all patients. Our data illustrates that similar discrepancies between Phoenix and VITEK2 were found in third-generation cephalosporin-resistant Enterobacterales.

Several previous studies have investigated the accuracy of automated AST systems. In contrast to our study, several studies found that Phoenix actually overestimated susceptibility, although mostly in multidrug-resistant isolates. A study assessing the accuracy of the Phoenix automated system in isolates expressing clinically relevant resistance mechanisms found very major errors in gentamicin susceptibility for 1% of Enterobacterales*,* but in 8% of *P. aeruginosa* and tobramycin (10). Only 0.4% of Enterobacterales isolates were measured resistant by Phoenix and susceptible with the reference method (the same Phoenix measurements but using CLSI breakpoints), and there were none of these errors for *P. aeruginosa*. However, since both measurements in this study were done by Phoenix, this might be an underestimation. A more recent study concluded that Phoenix significantly overestimates susceptibility to aminoglycosides for carbapenem-resistant isolates compared with broth microdilution (11). In another study, both VITEK2 and Phoenix were found to be suboptimal for aminoglycoside susceptibility testing for carbapenem-resistant Enterobacterales (12). In *Acinetobacter baumanii*, VITEK2 incorrectly reported that more than one-third of the isolates were susceptible to amikacin compared to microdilution (13). Although Phoenix was more accurate than VITEK2, very major error rates for aminoglycosides exceeded the levels allowable by the CLSI in both systems. Since these were mostly older studies, it is possible that the composition of the Phoenix and VITEK2 cards was different from today, leading to opposite results compared to our study. Importantly, none of these studies specifically looked at differences in the borderline susceptible area, and none looked at temporal changes.

Our study included data from a large nationwide surveillance database for antimicrobial resistance, which is rigorously checked for data quality. It includes a large number of participating MMLs using different automated testing systems, which makes it possible to recognize differences in measurements between systems. There are some limitations to this study. In addition to the automated testing system itself, the applied test panels might also play a role in the differences and changes over the years in MIC distributions. Unfortunately, there was no information available on the test panels which had been applied by the MMLs. Furthermore, since BD updated the Phoenix cards, the investigated isolates could not be re-tested in both automated systems.

This study shows that there might be an overestimation of the borderline susceptible and resistant isolates in MMLs that use a Phoenix system (at least until the update of the test panel). These findings should be taken into consideration in the development of clinical treatment guidelines for patients with sepsis and could play a role in the decision on the optimal dosage of aminoglycosides in the empirical treatment of patients with sepsis. A definitive answer as to whether a lower or higher dose of aminoglycosides is warranted in patients with sepsis (balancing efficacy and side effects) should be obtained by a clinical trial, which will be very costly and time-consuming. It might be very useful to create an (inter)national infrastructure where observations of sudden important changes in AST can be reported and interpreted and which can initiate and coordinate appropriate actions.
